# High-intensity decreasing interval training (HIDIT) increases time above 90% $\dot{V}$O_2_peak

**DOI:** 10.1007/s00421-020-04463-w

**Published:** 2020-08-11

**Authors:** Filippo Vaccari, N. Giovanelli, S. Lazzer

**Affiliations:** 1 Department of Medicine, University of Udine, P.le Kolbe 4, 33100 Udine, Italy; 2 School of Sport Sciences, University of Udine, Udine, Italy

**Keywords:** $\dot{V}$O_2_max, $\dot{V}$O_2_max training, Time at $\dot{V}$O_2_max, HIIT

## Abstract

**Purpose:**

Training near $\dot{V}$O_2_max is considered to be the most effective way to enhance $\dot{V}$O_2_max. High-intensity interval training (HIIT) is a well-known time-efficient training method for improving cardiorespiratory and metabolic function and $\dot{V}$O_2_max. While long HIIT bouts allow $\dot{V}$O_2_max to be achieved quickly, short HIIT bouts improve time to exhaustion (Tlim). The aim of this study was to evaluate the time spent above 90% $\dot{V}$O_2_peak (*T* > 90% $\dot{V}$O_2_peak) during three different HIIT protocols.

**Methods:**

Twelve cyclists performed three HIIT sessions. Each protocol had the same work and recovery power and ratio of work·recovery^−1^. The protocols consisted of long-interval HIIT (LI_HIIT_, 3 min work—2 min recovery), short-interval HIIT (SI_HIIT_, 30 s work—20 s recovery), and high-intensity decreasing interval training (HIDIT, work from 3 min to 30 s and recovery from 2 min to 20 s). *T* > 90% $\dot{V}$O_2_peak, Tlim, blood lactate [La], and rate of perceived exertion (RPE) were measured at Tlim.

**Results:**

*T* > 90% $\dot{V}$O_2_peak was greater in HIDIT (312 ± 207 s) than in SI_HIIT_ (182 ± 225 s; *P* = 0.036) or LI_HIIT_ (179 ± 145 s; *P* = 0.027). Tlim was not significantly different (*P* > 0.05) between HIDIT (798 ± 185 s), SI_HIIT_ (714 ± 265 s), and LI_HIIT_ (664 ± 282). At Tlim, no differences in [La] and RPE were found between protocols (*P* > 0.05).

**Conclusion:**

HIDIT showed the highest *T* > 90% $\dot{V}$O_2_peak, suggesting that it may be a good strategy to increase time close to $\dot{V}$O_2_peak, despite similar Tlim, [La], and RPE at Tlim.

## Introduction

Maximal oxygen uptake ($\dot{V}$O_2_max) refers to the oxygen consumption attained during a maximal exercise. It is reached when the $\dot{V}$O_2_ does not increase any further despite further increases in workload, and it defines the limits of the cardiorespiratory system (Hill and Lupton 1923). $\dot{V}$O_2_max is a relevant parameter of cardiorespiratory capacity, which is important for both endurance athletes (di Prampero 2003) and patients (Poole et al. 2012). It has been shown that, to improve $\dot{V}$O_2_max, a training protocol should prolong the time at which the oxygen uptake remains close to the maximum (within 5–10% of $\dot{V}$O_2_max) (Wenger and Bell 1986; Midgley and Mc Naughton 2006). High-intensity interval training (HIIT) is very effective at maintaining the metabolic rate near $\dot{V}$O_2_max (Buchheit and Laursen 2013a), better than continuous steady-state training (Midgley and Mc Naughton 2006), and can be comprised of either short or long bouts of high intensity (work) alternated with recovery periods (recovery) at low intensity (or rest) (Buchheit and Laursen 2013a).

The minimum intensity that allows one to reach $\dot{V}$O_2_max during a steady-state exercise is called critical power (CP). Theoretically, it is possible to maintain a metabolic steady state and prolong effort up to the CP threshold indefinitely. In contrast, above the CP, even if the external power output remains constant, $\dot{V}$O_2_ increases up to $\dot{V}$O_2_max, leading to exhaustion within a few minutes (Jones and Vanhatalo 2017).

HIIT can be set based on CP, setting the work intervals above CP and the recovery intervals below CP (Morton and Billat 2004). The CP is mathematically defined as the power asymptote of the hyperbolic relationship between power output and time to exhaustion (Jones et al. 2010). Physiologically, CP represents the boundary between steady-state and non-steady-state exercise intensity domains (Jones et al. 2010; Jones and Vanhatalo 2017). Exercise above CP leads to reduced muscle phosphocreatine concentration [Pcr] and pH (Meyer 1988; Chidnok et al. 2013; Jones and Vanhatalo 2017), making it difficult to prolong exercise (i.e., *W*′: amount of work that can be done during exercise above CP) (Ferguson et al. 2010; Skiba et al. 2012, 2014, 2015). Since muscle $\dot{V}$O_2_ is related to muscle reduction [Pcr] (di Prampero and Margaria 1968; Meyer 1988), the faster [Pcr] is depleted, the faster the $\dot{V}$O_2_ increases. Conversely, during the recovery phase (below CP), [Pcr] resynthesis and *W*′ recovery follow an exponential trend (Meyer 1988; Ferguson et al. 2010; Skiba et al. 2012, 2014; Jones and Vanhatalo 2017; Vinetti et al. 2017). Indeed, when exercise generates a large depletion of [Pcr], the resynthesis rate is faster in the beginning of the recovery and it slows when approaching complete restoration.

Thus, an HIIT protocol that aims to stimulate $\dot{V}$O_2_max should start with long work intervals (2–4 min) to quickly increase $\dot{V}$O_2_. Subsequently, when the subject approaches exhaustion, short intervals can help to prolong the exercise for longer: in this situation, the recovery ratio is fast and requires only few seconds to ensure sufficient recovery while simultaneously preventing the $\dot{V}$O_2_ from decreasing too much.

Therefore, the aim of this study was to compare the time above 90% of $\dot{V}$O_2_peak (*T* > 90% $\dot{V}$O_2_peak) in three different HIIT protocols. The proposed HIIT protocols had the same intensity and work/recovery ratio and were structured as follows: (1) constant long intervals (LI_HIIT_); (2) decreasing interval duration (high-intensity decreasing interval training, HIDIT), and (3) constant short intervals (SI_HIIT_). It has been hypothesized that the *T* > 90% $\dot{V}$O_2_peak should be longer in HIDIT. We hypothesized that the protocol with longer intervals followed by shorter intervals would elicit longer time above 90%.

## Materials and methods

### Subjects

Twelve middle-age amateur cyclists, all non-smokers, were enrolled in the study (41 ± 11 years; 76 ± 10 kg; $\dot{V}$O_2_peak 4.32 ± 0.47 L min^−1^), Table 1. They reported at least three training sessions per week in the previous 6 months. None of the subjects had evidence of significant diseases or took regular medications.

**Table 1:** Descriptive characteristics of the participants (*n* = 12)

	Mean ± SD	Min–Max
Age (year)	41 ± 11	29–62
Body mass (kg)	76 ± 10	66–95
HRpeak (b min^−1^)	174 ± 10	155–193
$\dot{V}$O_2_peak (L min^−1^)	4.32 ± 0.47	3.66–5.10
Load peak (W)	356 ± 40	295–436
CP (W)	254 ± 30	212–320
*W*' (kJ)	12.8 ± 4.1	8.5–22.7
High intensity (W)	297 ± 35	249–364
Low intensity (W)	212 ± 30	172–275

All values are mean and standard deviation (SD)

*HR* heart rate, $\dot{V}$*O*_*2*_*peak* peak oxygen consumption, *CP* critical power, *W*′ total work sustainable above critical power, *High and Low intensity* the average intensity sustained during HIIT tests

### Study protocol

The Ethics Committee of the Friuli-Venezia-Giulia approved the study (protocol number 9626). During the first visit to the laboratory, an operator explained the purposes and objectives of the study to each subject and obtained written informed consent. Then, participants underwent medical examinations and performed a maximal ramp-incremental exercise test on a cycle ergometer to measure the $\dot{V}$O_2_peak. Although the objectives were explained to all subjects, the study hypothesis was not revealed so as not to influence the results. After the first visit, the participants were examined three or four times to determine the critical power, and they performed the SI_HIIT,_ HIDIT, and LI_HIIT_ tests three times. Every visit was separated from the previous one by 2 days. Participants were instructed to avoid the consumption of caffeinated beverages for at least 8 h before each test and to abstain from vigorous physical activity in the 24 h preceding each testing session. Every subject concluded the entire protocol within 4 weeks from the first visit. The critical power parameters were used to program the HIIT tests. Subsequently, during the three HIIT tests, time to exhaustion (Tlim), *T* > 90% $\dot{V}$O_2_peak, blood lactate concentration [La], rate of perceived exertion using the Borg CR10 Scale (Borg et al. 2010), and $\dot{V}$O_2_ were measured at the 3rd minute and at the end of exercise.

### Incremental exercise

The incremental exercise was performed under medical supervision, and standard safety procedures were followed. During the first visit, an operator instructed the subjects to correctly report the rate of perceived exertion on the CR10 scale (Borg et al. 2010). The incremental exercise, critical power trials, and HIIT test protocols were performed utilizing a cycle ergometer (CE) (Monark Ergomedic 839E). Every test was preceded by the same warm-up procedure: 10 min cycling at 100 W followed by 2-min resting. During the first warm-up, subjects chose their preferred pedaling cadence (~ 90 rpm). The incremental exercise was a constant incremental ramp test started at 100 W and gradually increased by 1 W every 2.4 s (25 W min^−1^) throughout the test until voluntary exhaustion. The exhaustion (during the incremental test and the HIITs) was defined as the inability to maintain the assigned cadence within 10 rpm longer than 5 s despite strong encouragement from the operator.

$\dot{V}$O_2_ and $\dot{V}$CO_2_ were measured breath-by-breath using a metabolic unit (Quark CPET, Cosmed, Italy). The ventilation was measured by a turbine calibrated before each test with a 3-L syringe at three different flow rates. Calibration of O_2_ and CO_2_ analysers was performed before each test by utilizing calibration gas mixtures of known composition (16.00% O_2_; 4.00% CO_2_). $\dot{V}$O_2_peak corresponded to the highest mean $\dot{V}$O_2_ obtained in 30 s at the end of the incremental exercise.

### Power–duration relationship

The same warm-up and cadence from the incremental test were also used for the critical power (CP) test. CP and the amount of work that could be done during exercise above CP (*W*′) (Jones and Vanhatalo 2017; Burnley and Jones 2018) were estimated from three to four high-intensity trials at exhaustion from 80 to 100% of the peak power detected during the incremental test and adopted to result in ‘exhaustion’ in a minimum of ~ 2 min and a maximum of ~ 15 min (Jones and Vanhatalo 2017). The work done in each of the separate exercise bouts has been plotted against Tlim. The following work (W) − time (t) linear regression was then used to find CP and *W*′ (Moritani et al. 1981; Hill 1993; Jones and Vanhatalo 2017):

1 $$W = CPt + W^{\prime}.$$

According to the equation, CP is given by the slope of the regression, and the *W*′ is the *y*-intercept.

### HIIT tests

After the incremental test and the critical power trials, subjects performed three HIIT tests in a randomized order. The power during the work and recovery bouts and the work/recovery duration ratio were the same in each trial, although the duration of the intervals was changed (see Table 1 for mean values). The ratio work/recovery time was set at 3/2 for all the training tests. The power used for the high-intensity bouts was customized for each subject and corresponded to the power that was supposed to lead to exhaustion in 5 min (300 s) according to the following equation (Jones et al. 2010):

2 $${\text{Power}} = \frac{{W^{\prime}}}{{t = 300\;{\text{s}}}} + {\text{CP,}}$$

and it corresponded to approximately 117% of CP. The power used for the low-intensity bout was mirrored below CP (approximately 83% of CP). Thus, the CP threshold was exactly in the middle between the high and low intensities.

**Fig. 1:**
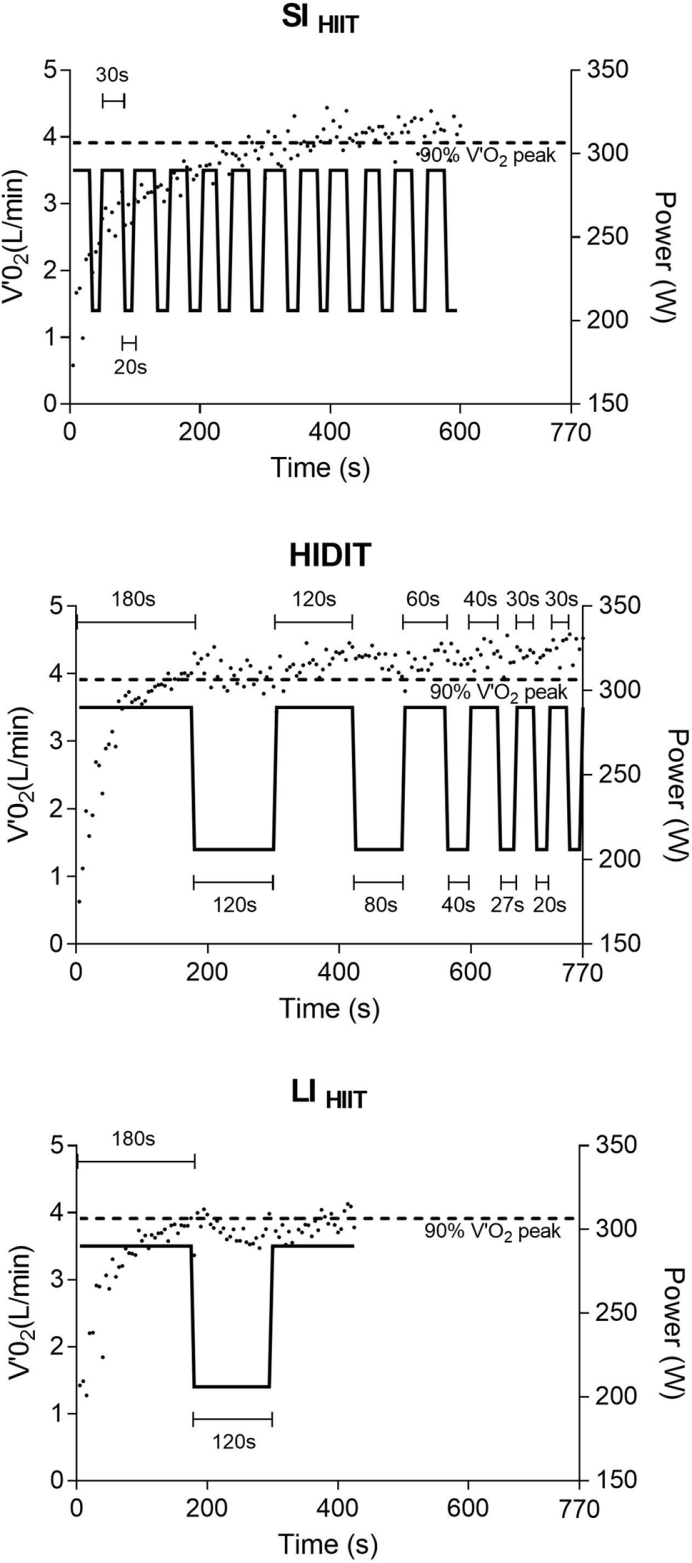
HIIT protocols for a representative subject. SI_HIIT_: short-interval HIIT (30″ high—20″ low-intensity); HIDIT: decreasing intervals HIIT (combining high intensity from 3′ to 30″ and low intensity from 2′ to 20″); LI_HIIT_: long-interval HIIT (3′ high—2′ low-intensity); the dotted lines represent the breath-by-breath $\dot{V}$O_2_ data averaged every 5 s; the dashed lines represent the threshold of 90% of $\dot{V}$O_2_peak; the solid lines represent the actual power

The three tests were structured as follows (Fig. 1):

- Short intervals (SI_HIIT_): 30 s at high intensity and 20 s at low intensity repeated until volitional exhaustion of the subject.
- High-intensity decremental interval training (HIDIT): 3 min at high intensity and 2 min at low intensity; 2 min at high intensity and 1 min and 20 s at low intensity; 1 min at high intensity and 40 s at low intensity; 45 s at high intensity and 30 s at low intensity; and finally 30 s at high intensity and 20 s at low intensity, repeated until volitional exhaustion of the subject. The high–low ratio intensity duration was always 3/2.
- Long intervals (LI_HIIT_): 3 min at high intensity and 2 min at low intensity repeated until volitional exhaustion of the subject.

Throughout the HIIT protocols, the ventilatory parameters were measured using a breath-by-breath metabolic unit (CPET, Cosmed, Italy) and then averaged every 5 s. Before, after 3 min and at the end of exercise, $\dot{V}$O_2_, HR, [La], and RPE were measured, and the respiratory quotient (RQ) was calculated. An operator collected a capillary blood sample from the earlobe to measure the [La] with a dedicated device (Lactate Pro 2, Arkaray Inc., Japan), while the subjects reported RPE consulting the CR10 scale positioned in front of them. Finally, the total time spent above 90% of $\dot{V}$O_2_peak was determined as the sum of each averaged 5-s when the $\dot{V}$O_2_ was equal to or higher than 90% of $\dot{V}$O_2_peak.

### Statistical analyses

Statistical analysis was performed using SPSS 20.0 software (IBM, Chicago, USA) with significance set at *P* < 0.05. All results were expressed as the means and standard deviations (SD). The differences between HIIT training protocols in Tlim; *T* > 90% $\dot{V}$O_2_peak; *T* > 90% $\dot{V}$O_2_peak—Tlim^−1^; work above CP (calculated as the total time in seconds above CP multiply by the difference between the high-intensity power and CP, in Watts); average $\dot{V}$O_2_; and, finally, the values at the third minute and at Tlim ($\dot{V}$O_2_, HR, [La], CR10-scale and RQ) were investigated. All parameters were analyzed by one-way repeated-measures analysis of variance (ANOVA). Where the analysis found a significant difference, planned contrast between HIDIT and SI_HIIT_ and between HIDIT and LI_HIIT_ were used with Bonferroni correction to determine the origin of such effects. The confidence intervals (CIs) of the differences and the effect size (ES) were calculated using Cohen’s *d* (0 < *d* < 0.20, small; 0.20 < *d* < 0.50, medium; *d* > 0.50, large) (Cohen 1988). The precision of Cp and *W*′ estimation was calculated comparing the parameter estimates with the work-time model and with the time^−1^ model through a *t* test. For our purposes, a sample size of 12 subjects was calculated to have a statistical power of 80% to refute the null hypothesis and to obtain an ES of 0.88 with an alpha error of 0.05 and a beta error of 0.20 using a one-way ANOVA with Bonferroni correction, according to a previous study (De Aguiar et al. 2013) that implemented a procedure similar to that of our study.

## Results

### Incremental test and CP trials

Peak values attained during the incremental test, CP, total work above CP (*W*′), and the power imposed for the high- and low-intensity bouts are shown in Table 1. Although the attainment of $\dot{V}$O_2_peak was not set as a priori criteria for the constant work rate tests of the power–duration relationship, it was always reached by the subjects. The parameter estimates through the “work-time model” used for our purposes have been compared with the parameter estimates through the “1·time^−1^” model, and the results were comparable, as shown in Table 2.

**Table 2:** Comparison of the power–duration relationship derived from 1/time model CP and work-time model CP

Subject	Critical power estimates		*W*' estimates		*R*^2^	
	1/Time model CP (W)	Work-time model CP (W)	1/Time model *W*′ (kJ)	Work-time model *W*′ (kJ)	1/Time model	Work-time model
1	212	217	11.9	11.2	0.966	0.997
2	259	262	9.9	9.5	0.999	0.994
3	221	225	8.5	7.8	0.999	0.942
4	254	252	12.8	13.3	1.000	0.997
5	278	278	9.9	9.9	1.000	1.000
6	248	240	12.5	14.2	0.996	0.956
7	256	255	8.0	8.1	0.999	1.000
8	320	317	13.3	14.0	0.999	0.993
9	258	258	13.7	13.8	1.000	1.000
10	280	275	22.7	24.9	0.999	0.981
11	223	223	18.1	18.2	0.999	1.000
12	243	240	12.2	13.0	0.997	0.984
Mean	254	254	12.8	13.2	0.996	0.987
Standard deviation	30	28	4.1	4.7	0.010	0.019
*t* test		0.456		0.178		0.183

*R*^*2*^ coefficient of determination of the linear regression, *CP* critical power, *W′* total work sustainable above the critical power

Student paired *t* test: no significant differences between the parameters of the power–duration relationship derived from the two different CP models were observed

### HIIT tests

The power corresponding to high-intensity intervals was 117 ± 6% of CP, and the low-intensity power was 83 ± 6% of the CP (Table 3).

**Table 3:** Main results of the HIIT tests and selected physiological variable at 3rd minute and at the end of the tests

	SI_HIIT_	HIDIT	LI_HIIT_	*P*
Tlim (s)	714 ± 265	798 ± 185	664 ± 282	0.144
*T* > 90% $\dot{V}$O_2_peak (s)	183 ± 225	312 ± 207^a,b^	179 ± 145	0.029
*T* > 90% $\dot{V}$O_2_peak × Tlim^−1^	0.25 ± 0.29	0.39 ± 0.24	0.26 ± 0.21	0.070
Work > CP (KJ)	18.74 ± 8.95	22.01 ± 10.40	19.28 ± 11.06	0.136
Mean %$\dot{V}$O_2_peak	81.50 ± 6.61	84.16 ± 4.00^b^	79.58 ± 7.08	0.044
Values at 3rd minute				
%$\dot{V}$O_2_peak	85.33 ± 7.11	90.75 ± 5.94^a^	89.58 ± 6.52	0.004
%HRpeak	89.00 ± 4.00	91.00 ± 3.91^a^	92.60 ± 3.60	0.003
[La] (mmol L^−1^)	5.69 ± 1.62	8.03 ± 2.69^a^	7.85 ± 3.01	0.007
CR10-scale	5.29 ± 1.57	6.67 ± 2.12^a^	6.52 ± 2.03	0.008
RQ	1.04 ± 0.06	1.10 ± 0.09^a^	1.11 ± 0.08	> 0.001
Tlim				
%$\dot{V}$O_2_peak	99.75 ± 8.62	100.17 ± 5.27	99.83 ± 8.36	0.981
%HRpeak	97.80 ± 3.99	97.40 ± 2.99	97.50 ± 3.98	0.802
[La] (mmol L^−1^)	10.75 ± 2.04	10.71 ± 4.72	10.83 ± 3.58	0.991
CR10-scale	9.48 ± 0.70	9.25 ± 1.78	9.56 ± 1.08	0.701
RQ	0.97 ± 0.05	0.95 ± 0.05	1.00 ± 0.10	0.113

All values are mean and standard deviation (SD)

*SI*_*HIIT*_ short-interval HIIT, *HIDIT* high-intensity decremental intervals training, *LI*_*HIIT*_ long-interval HIIT, *Tlim* time to exhaustion, *T > 90%*$\dot{V}$*O*_*2*_*peak* time spent above 90% $\dot{V}$O_2_peak, *%*$\dot{V}$*O*_*2*_*peak* oxygen uptake in percentage relative to its peak, *mean%*$\dot{V}$*O*_*2*_*peak* mean %$\dot{V}$O_2_peak maintained during HIIT tests, *%HRpeak* heart rate in percentage relative to its peak, *[La]* blood lactate concentration, *CR10-scale* perceived exertion, *RQ* respiratory quotient

Significance by one-way repeated-measure ANOVA. When *P* < 0.05, planned contrasts with Bonferroni correction

^a^*P* < 0.05 in post hoc HIDIT vs SI_HIIT_

^b^*P* < 0.05 in post hoc HIDIT vs LI_HIIT_

**Fig. 2:**
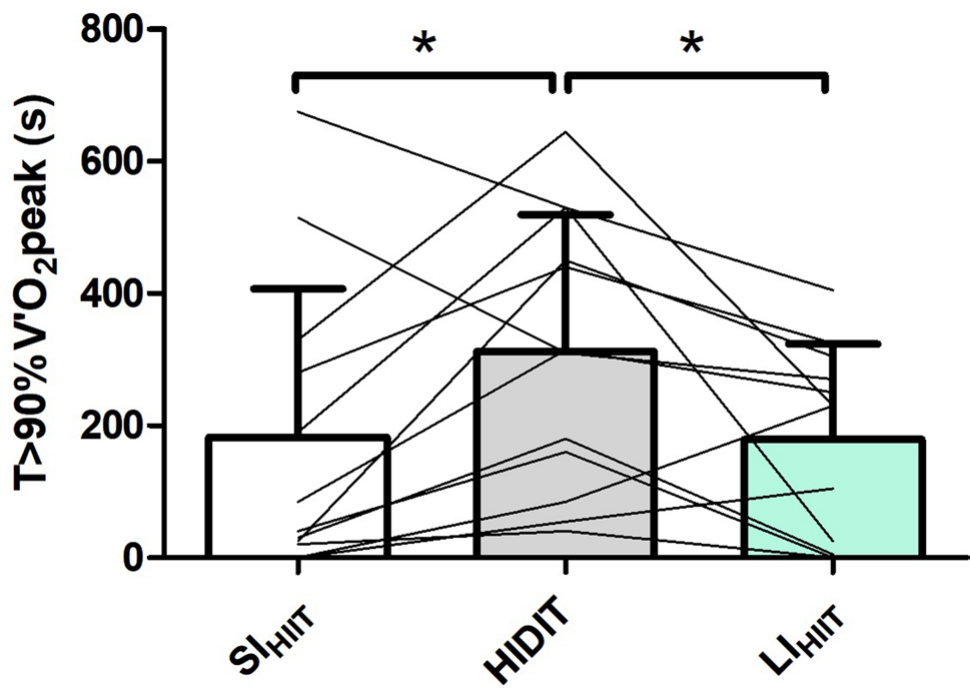
Time above 90% of $\dot{V}$O_2_ peak in seconds. *Significance by one-way repeated-measures ANOVA and planned contrast with Bonferroni correction between HIDIT and SI_HIIT_ and between HIDIT and LI_HIIT_ were used post hoc comparison, *P* < 0.05

T > 90% $\dot{V}$O_2_peak was significantly longer in HIDIT compared with SI_HIIT_ (*P* = 0.036; ES: 0.62) and LI_HIIT_ (*P* = 0.027; ES: 0.64) (Table 3, Fig. 2), and the ratio *T* > 90% $\dot{V}$O_2_peak—Tlim^−1^ tended to be higher in HIDIT than in SI_HIIT_ and LI_HIIT_ (Table 3). However, there were no differences in Tlim and in work > CP (*P* = 0.136) between the three protocols (Table 3). Finally, the average $\dot{V}$O_2_ maintained during the HIDIT test was significantly higher than in LI_HIIT_ (*P* = 0.022; ES: 0.17) but not significantly different than in SI_HIIT_ (*P* = 0.106; ES: 0.10).

% $\dot{V}$O_2_peak after 3 min was similar between HIDIT and LI_HIIT_ (*P* = 0.339; ES: 0.18), but it was significantly higher in HIDIT than SI_HIIT_ (*P* = 0.006; ES: 0.83) (Table 3). Additionally, %HRpeak after 3 min was similar between HIDIT and LI_HIIT_ (*P* = 0.160; ES: 0.37), but it was significantly higher in HIDIT compared with SI_HIIT_ (*P* = 0.019; ES: 0.61). Similarly, the CR10-scale after 3 min was similar in HIDIT and LI_HIIT_ (*P* = 0.824; ES: 0.05) but significantly higher than SI_HIIT_ (*P* = 0.031; ES: 0.55). Finally, RQ after 3 min was not significantly different in HIDIT and LI_HIIT_ (*P* = 0.410; ES: 0.05), but it was significantly higher than in SI_HIIT_ (*P* = 0.031; ES: 0.25) (Table 3).

There was no significant difference in [La] at rest before the three tests (SI_HIIT_, HIDIT, and LI_HIIT_) (1.13 ± 0.20; 1.19 ± 0.26; and 1.17 ± 0.27 mmol L^−1^, respectively; *P* > 0.05), and after 3 min, [La] was similar in HIDIT and LI_HIIT_ (*P* = 0.007; ES: 0.05), but lower in SI_HIIT_ (*P* = 0.003; ES: 0.78) (Table. 3). At Tlim, neither [La] nor $\dot{V}$O_2_, HR nor RPE were significantly different between the three tests (see Table 3).

## Discussion

The results of the present study show that a new HIDIT protocol maintains the $\dot{V}$O_2_ above 90% of $\dot{V}$O_2_peak for a longer period compared with two classical HIIT protocols with short and long intervals. Nevertheless, the Tlim, [La], HR, RPE, and $\dot{V}$O_2_ were similar among the protocols. This is the first study that has demonstrated that it is possible to increase the time close to $\dot{V}$O_2_peak solely through decreasing the duration of the intervals and, therefore, avoiding reducing the power/intensity as previously shown (De Aguiar et al. 2013; Lisbôa et al. 2015; Rønnestad and Hansen 2016).

In HIDIT (and LI_HIIT_), the protocol begins with 3 min at high intensity, as opposed to just 30 s in SI_HIIT_, and this resulted in a greater $\dot{V}$O_2_, HR, [La], CR10 scale, and RQ after 3 min of exercise. This is consistent with the studies by Millet et al. (2003) and Turner et al. (2006), in which during long-interval HIIT, a faster metabolic stimulation occurred at the beginning of the cycling exercise. However, in our study, there were no differences at Tlim in any of the parameters mentioned above, suggesting that the participants reached their personal maximal performances, regardless of the protocol adopted. Indeed, $\dot{V}$O_2_ and HR were close to the peak values (100% and 97%, respectively), while Borg scale was near 10 and [La] was above 10 mmol L^−1^. It is worth noting that HIDIT led to longer *T* > 90% $\dot{V}$O_2_peak despite the same RPE at the end of the exercise. In other words, HIDIT has potentially better training benefits, despite the same perceived effort. On the other hand, even though Tlim in HIDIT (798 s) was longer than in LI_HIIT_ (664 s) and, similar to SI_HIIT_, (714 s), the ANOVA did not show any significant difference (*P* = 0.144). Our results seem to contradict results from the previous studies (Millet et al. 2003; Turner et al. 2006; Rønnestad and Hansen 2016). Millet et al. (2003) showed that when comparing some matched work HIIT protocols, those with shorter intervals elicited lower $\dot{V}$O_2_, HR, and RPE at the end of the exercise, suggesting that the duration may be longer when shorter intervals are used. Similarly, Turner et al. (2006) compared four HIIT protocols with the same intensity (work and recovery) and work/recovery ratio, reporting that in HIIT with shorter intervals, the [La] was lower after 30 min of exercise compared with longer intervals. In particular, in the HIIT protocol with shorter intervals (work 10 s/recovery 20 s), the [La] reached steady state after 30 min of exercise, whereas the one with longer intervals (work 90 s/recovery 180 s), the subjects lasted less than 10 min before exhaustion.

Surprisingly, there are a few studies in which the authors analyze the effects of interval duration at a fixed work/recovery ratio and a fixed intensity (Millet et al. 2003; Turner et al. 2006; Rønnestad and Hansen 2016). It is known that increasing work interval durations prolongs the time close to $\dot{V}$O_2_max (Rozenek et al. 2007; Wakefield and Glaister 2009). Conversely, longer recovery interval duration decreases the time close to $\dot{V}$O_2_max (Smilios et al. 2017). However, to our knowledge, the only study that measured the time close to $\dot{V}$O_2_max and Tlim in HIIT matching work rate and work/recovery ratio and isolating the interval duration variable was performed by Rønnestad and Hansen (Rønnestad and Hansen 2016). They compared three cycling HIIT protocols in which the intensity of the work bouts was set at maximal aerobic power ($\dot{V}$O_2_max power), the recovery at 50% of the $\dot{V}$O_2_max power, and the work/recovery ratio was 2/1. They concluded that HIIT with shorter interval durations (30 s) led to a longer Tlim (~ 1400 s), a longer Time > 90% $\dot{V}$O_2_peak (~ 680 s) and a higher ratio of Time > 90% $\dot{V}$O_2_peak·Tlim^−1^ (0.55) (Rønnestad and Hansen 2016). Tlim, Time > 90% $\dot{V}$O_2_peak, and their ratio were lower in our study. This discrepancy may be attributed to the different protocols used and to the higher fitness level of the participants ($\dot{V}$O_2_peak = 66 mL kg^−1^ min^−1^ compared to 57 mL kg^−1^ min^−1^) (Rønnestad and Hansen 2016). Another possible explanation might be the relative intensity at which our protocol was set (on average ~ 83% of load peak). This relative intensity refers to the load peak attained during a ramp protocol, which is reported to be 10–15% higher than the load peak reached with a step modality (Revill et al. 2002; Bentley and McNaughton 2003; Zuniga et al. 2012). Therefore, it can be assumed that the relative power would have been above 90% of the load peak if the incremental test was performed using steps. Nevertheless, the incremental ramp test was used alone in the present study only to determine $\dot{V}$O_2_peak, while the intensity of HIIT was set exclusively considering CP, as described above.

In an attempt to benefit from faster $\dot{V}$O_2_ kinetics at the beginning of exercise, we imposed long first intervals. Alternately, other authors proposed a fast start strategy (De Aguiar et al. 2013; Lisbôa et al. 2015; Rønnestad et al. 2019). Fast start strategy HIIT protocol (starting from 125% of the intermittent critical power, ICP, and decreasing it until 105%) enhanced the time above 95% of $\dot{V}$O_2_max compared to other protocols with a constant work rate at 125% ICP and a constant work rate at 105% ICP (De Aguiar et al. 2013). Nevertheless, the protocol that used lower intensity (105% ICP) increased Tlim, and the protocol that adopted higher intensity bouts (125% ICP) showed a greater ratio of Tlim/time above 95% of $\dot{V}$O_2_max^−1^. Lisbôa et al (2015) decreased the intensity within every single interval, but attained similar results. In addition, the recent work of Rønnestad et al. (2019) confirmed that the fast start pacing strategy can be a good strategy to increase the average $\dot{V}$O_2_, but the time close to $\dot{V}$O_2_max was not longer compared to traditional HIIT. Therefore, the fast start strategy is a useful tool to improve time near/at $\dot{V}$O_2_max and could be successfully applied to HIIT, although it impairs Tlim in comparison with protocols with the same final exercise work rate and the ratio *T* > 90% $\dot{V}$O_2_peak − Tlim^−1^ in comparison with protocols with the same initial intensity (De Aguiar et al. 2013). Compared to fast start protocols, HIDIT has the advantage of quickly stimulating oxygen uptake at the beginning without affecting Tlim. Moreover, fast start strategy HIIT reduces the ratio *T* > 90% $\dot{V}$O2peak—Tlim^−1^, while HIDIT tends to increase it (not significantly). Therefore, the HIDIT protocol that this study proposed combines the advantages of different previously studied protocols and can be used during training sessions that aim to accumulate time close to $\dot{V}$O_2_max.

Nonetheless, it is interesting that several participants were able to drastically increase the *T* > 90%VO_2_peak in the HIDIT protocol, whereas others performed much worse. In addition, as discussed above, the ANOVA failed to find differences in Tlim between the three HIIT protocols, which could be due to the heterogeneity of the subjects, despite our efforts to minimize differences by setting up HIIT reliant on CP and *W*′. In fact, high intensity was set as the percentage of CP that allowed each subject to last for 5 min before exhaustion according to equation [2]. While the intensity of HIIT is often set relying on %$\dot{V}$O_2_max, relying exclusively on $\dot{V}$O_2_max does not take into account the anaerobic characteristics of the subjects, which are very important in HIIT. For instance, whether two athletes present a similar $\dot{V}$O_2_max intensity but different *W*′ (and CP) when exercising with similar %$\dot{V}$O_2_max intensity during HIIT, the exercise will actually involve a different proportion of their *W*′, which results in a different exercise tolerance (Blondel et al. 2001). Therefore, expressing intensity as a percentage of CP for high-intensity exercises allows individual differences in *W*′ to be taken into account and eased as much as possible. Indeed, *W*′ was not correlated with Tlim of any HIIT test, since it has been used to adjust the intensity with equation [2]. Furthermore, there was no correlation among age/HRpeak, the $\dot{V}$O_2_ kinetics during the first 3 min of HIDIT and LI_HIIT_ (unpublished), and the other main outcomes. Additionally, there were no relationships between $\dot{V}$O_2_peak or CP and the main outcomes as well. The lack of relationship among age and other variables suggests that age did not influence our main results. In fact, our data may even support the idea that HIDIT could be applied in well-trained male adults over a wide range of age. Another major physiological determinant that may explain the variability between subjects in Tlim during interval and continuous exercises is the differences between lactate threshold intensity and $\dot{V}$O_2_max intensity (Midgley et al. 2007). Midgley et al. suggested that athletes with larger differences will replete their anaerobic capacity to a greater extent during each relief interval, increasing the time to exhaustion. Similarly, the relationship between the CP-load peak difference and Tlim during HIIT has been verified in this study to determine whether it can affect the Tlim of HIIT. As a result, only 59% of the variance in Tlim in SI_HIIT_ was explained by the difference between CP and load peak in percentage, while in the other two protocols, there were no relationships. Therefore, future research that aims to investigate Tlim in HIIT may benefit by selecting subjects with homogeneous difference %CP-load peak, although Tlim in HIIT with longer intervals does not seem to correlate with it. It is, therefore, tempting to suggest that individuals with a wide gap between the CP and the load peak could benefit more from short-interval HIIT to prolong Tlim.

Further research is needed to verify whether *T* > 90% $\dot{V}$O_2_peak may be enhanced with HIDIT in different HIIT protocols (i.e., at different intensities) and in different populations. However, HIDIT might be useful in sport training when the aim is to maintain a high $\dot{V}$O_2_max and/or maintain a specific power or velocity as long as possible, such as in training for track cycling races. If the aim is to allow the athlete to finish the race at a given time, the most specific training is to ride at that velocity for that race time for a distance as near as possible to the distance of the race. After the recovery, repeat for a shorter distance and so on. Starting with short intervals would not be sufficiently specific, and continuing with the first interval distance would not be possible for the fatigued athlete.

Furthermore, HIDIT could be useful for patients or for wellness purposes, setting a lower percentage of $\dot{V}$O_2_max or other physiological parameters. For example, if an exercise is intended to avoid exceeding a given [La] cut-off, it can start with a longer interval to save time and then decrease the length of the interval to avoid exceeding the [La] cut-off. However, we suggest adopting this protocol in athletes and patients who aim to train and improve their $\dot{V}$O_2_max.

## Conclusions

In conclusion, HIDIT applied to cycling exercise in well-trained amateur cyclists can enhance *T* > 90% $\dot{V}$O_2_peak without reducing Tlim, the ratio of *T* > 90% $\dot{V}$O_2_peak and Tlim^−1^, or the average $\dot{V}$O_2_. In fact, the average $\dot{V}$O_2_ was even higher in HIDIT than in LI_HIIT_. Finally, despite the higher stimulation of $\dot{V}$O_2_, the rate of perceived exertion and the other physiological parameters at the end of the exercise were not different compared with long- or short-interval HIIT, suggesting that HIDIT was not more demanding. In light of the favorable or similar physiological and/or perceptual responses to HIDIT compared to the other protocols and given the improved capability to prolong the time close to $\dot{V}$O_2_peak, it could be used as a preferable method to elicit similar or greater physiological adaptations.

## Abbreviations

%CP-Load Peak: Percentage of critical power relative to load peak
%$\dot{V}$O_2_peak: Oxygen consumption in percentage relative to its peak
%HRpeak: Heart rate in percentage relative to its peak
[La]: Blood (capillary) lactate concentration
ANOVA: Analysis of variance
CP: Critical power
CR10 Scale: Validated scale of perceived exertion
ES: Effect size
HIDIT: Decreasing intervals HIIT (combining high phosphocreatine intensity from 3′ to 30″ and low intensity from 2′ to 20″)
HIIT: High-intensity interval training
ICP: Intermittent critical power
LI_HIIT_: Long intervals HIIT (3′ high—2′ low-intensity)
[Pcr]: Muscular concentration of phosphocreatine
QR: Gas-exchange ratio
RPE: Rate of perceived exertion
SI_HIIT_: Short intervals HIIT (30″ high—20″ low-intensity)
Tlim: (Time to exhaustion)
*T* > 90% $\dot{V}$O_2_peak: Time spent above 90% $\dot{V}$O_2_peak
$\dot{V}$CO_2_: CO_2_ output
$$\dot{V}$$O_2_: Pulmonary O_2_ uptake
$\dot{V}$O_2_max: Maximal theoretical aerobic power
$\dot{V}$O_2_peak: Maximal $\dot{V}$O_2_ achieved during incremental exercise
*W*′: Amount of work that can be done during exercise above CP
